# Prevalence and Associations of General Practice Registrars’ Management of Atopic Dermatitis: A Cross-Sectional Analysis from the Registrar Clinical Encounters in Training Study

**DOI:** 10.5826/dpc.1104a128

**Published:** 2021-10-01

**Authors:** Anneliese Willems, Amanda Tapley, Alison Fielding, Er Tsing Vivian Tng, Elizabeth G Holliday, Mieke L van Driel, Jean I Ball, Andrew R Davey, Irena Patsan, Kristen FitzGerald, Neil A Spike, Parker J Magin

**Affiliations:** 1 Eastern Victoria General Practice Training, Regional Training Organisation, Hawthorn, Victoria, Australia; 2 University of Melbourne, Department of General Practice and Primary Health Care, Berkeley Street, Carlton, Victoria, Australia; 3 University of Newcastle, School of Medicine and Public Health, University Drive, Callaghan, Newcastle, NSW, Australia; 4 GP Synergy, Regional Training Organisation, NSW & ACT Research and Evaluation Unit, 20 Mclntosh Drive, Mayfield West, NSW, Australia; 5 Department of Dermatology, John Hunter Hospital, Newcastle, New Lambton Heights, NSW, Australia; 6 The University of Queensland Faculty of Medicine, Primary Care Clinical Unit, Faculty of Medicine, Level 8 Health Sciences Building, Royal Brisbane & Women’s Hospital, Brisbane, QLD, Australia; 7 Hunter Medical Research Institute, Clinical Research Design, IT and Statistical Support Unit (CReDITSS), New Lambton, NSW, Australia; 8 University of Tasmania Tasmanian School of Medicine, Hobart, TAS, Australia; 9 General Practice Training Tasmania, Regional Training Organisation, Hobart, TAS, Australia

**Keywords:** Atopic dermatitis, eczema, general practice, dermatologists, family practice

## Abstract

**Introduction:**

Atopic dermatitis (AD) is a chronic inflammatory condition which imposes substantial burden upon patients and their families. As a frequent primary care presentation, general practice (GP) trainees must develop adequate skills in AD diagnosis and management.

**Objectives:**

We aimed to explore the prevalence and associations of GP registrars’ management of patients with AD.

**Methods:**

This study used data from the Registrar Clinical Encounters in Training (ReCEnT) project, an ongoing cohort study of the clinical and educational experience of Australian GP registrars. Registrar, patient, and consultation factors were independent variables in multivariable logistic regression with outcome factor ‘diagnosis/problem being AD’.

**Results:**

From 2010–2019, 2,783 registrars (96% response rate) provided data from 381,180 consultations. AD was encountered in 0.6% of consults. AD was more likely to be seen in patients aged 0–1 years and patients from a non-English speaking background. AD was less likely to be seen in Aboriginal or Torres Strait Islander patients. Learning goals were more likely to be generated for AD and these consultations were associated with registrars seeking information or assistance. AD was strongly associated with a medication being prescribed, of which the most prescribed medications were mild or moderate potency topical corticosteroids.

**Conclusions:**

Our findings suggest that, similar to other dermatological presentations, registrars find AD challenging to manage. There may be some gaps in AD management knowledge and application.

## Introduction

Atopic dermatitis (AD) is a chronic inflammatory skin condition[ 1] caused by the interaction of numerous environmental, genetic, and immune factors.[2] AD presents largely in childhood [3], with an estimated 12-month prevalence of 16–17% in childhood [4]. AD persists in teenagers and adults in approximately 50% of pediatric patients [5]. It is characterized by chronic inflammation and pruritis [6], and sufferers encounter a relapsing and remitting disease course [7]. Carrying the heaviest global burden of skin disease [8], AD is associated with significant physical and mental health sequelae, including effects on mood, sleep, and quality of life [7, 9, 10]. Childhood AD also profoundly impacts the financial, social, and psychological wellbeing of their families [11, 12].

A clinical diagnosis in most cases [1, 6], AD management depends on disease severity [13]. The majority of AD is mild [14, 15] and may be managed initially by patient education, emollients and, where appropriate, topical corticosteroids (TCS)[6]. For more severe disease, additional therapies including narrowband ultraviolet B phototherapy and oral immunosuppressive therapies may be considered through specialist consultation [16].

As a common primary health presentation [7, 17] general practice (GP) registrars may encounter AD throughout their training. In Australia, general practice training operates through an in-practice apprenticeship model [18–20]. Competency in the diagnosis and management of dermatological presentations, including AD, comprises an essential component of this training. Historically registrars have found dermatological presentations a challenging area [21].

While training seeks to provide a typical diagnostic cross-section of what their more senior counterparts see, variations in patients and problem exposure have been reported [19, 22, 23]. In particular, registrars see younger patients and have less exposure to chronic disease [19, 22, 24–26]. How this might translate to the registrar’s clinical exposure to AD has not been established yet. The aims of this study were to explore the prevalence and associations of GP registrars managing patients with AD.

## Methods

General practice training in Australia involves a minimum of three 6-month full-time-equivalent community-based general practice training terms. The Registrar Clinical Encounters in Training (ReCEnT) project records Australian GP registrar’s clinical and educational experience over these training terms. Data is collected once in each term, totaling 3 cycles of data collection. Registrars (vocational trainees in specialist general practice) complete a paper-based Case Report Form for each of 60 consecutive consultations.

Data recorded within these consultations includes patient demographics, diagnosis/problems, management, and referral choice. Problems/diagnoses are coded according to the International Classification of Primary Care (2^nd^ edition) classification system (ICPC-2 Plus) [27]. In addition, on a 6-monthly basis, registrar and practice variables are also collected via questionnaire.

From 2010–2015, ReCEnT was conducted in up to 5 Regional Training Providers (RTPs; across 5 states), prior to a major restructuring of Australian GP vocational training. From 2016, ReCEnT has been conducted in three Regional Training Organisations (RTOs), training 44% of Australia’s GP registrars,[28] across 3 states and a territory.

For the analyses presented in this report, data from 2010–2019 was included.

### Outcome Measure

The outcome variable was a problem/diagnosis of AD. Problems/ diagnoses coded as ‘dermatitis, atopic’, ‘eczema’ and ‘eczema, infantile’ were included within this outcome factor.

### Independent Variables

Independent variables considered in analyses encompassed registrar, patient, consultation, and practice factors (described below).

Registrar factors were registrar gender and age, the term of GP training, whether the registrars had worked at their current practice previously, country of primary medical degree (Australian versus international), and full-time or part-time employment status.

Practice factors were the rurality of the practice (using the Australian Statistical Geographical Classification - Remoteness Area (ASGC-RA) classification) [29], the size of the practice, whether the practice was fully bulk-billing (wherein no fee is charged to the patient), socio-economic position of the practice locality (based on Australian Socio-Economic Indexes for Areas-Index of Relative Socio-economic Disadvantage (SEIFA-IRSD)) [30], and the region in which the registrar was working.

Patient factors were patient gender, age group, non-English speaking background status, Aboriginal or Torres Strait Island status, and whether the patient was new to the practice, new to the registrar, or an existing patient.

Consultation Factors were the length of the consultation, whether AD was a new problem, the number of problems managed within the consultation, whether pathology was ordered, medications prescribed, and whether follow-up was arranged.

Educational consultation factors were whether sources of information (supervisor, electronic or hardcopy) were sought for the problem/diagnosis and whether learning goals were generated.

### Medications Prescribed

We also examined prescribing in more detail. Prescribed medications were tabulated for frequency of prescribing and, where possible for topical corticosteroids (TCS), the potency of the prescribed medication. TCS potency was inferred according to potency rankings[31] noting, where applicable, if that drug was available in different potency formulations.

### Statistical Analyses

The proportion of problems/diagnosis that were atopic dermatitis was calculated with 95% Confidence Intervals.

Descriptive statistics included frequencies with percent for categorical variables and mean with standard deviation (SD) for continuous variables. The frequencies of categorical variables were compared between outcome categories using Chi-squared tests or Fisher’s exact test when there was an expected count less than 5 in 25% or more cells). For continuous variables, means were compared using a t-test.

Univariable (simple) and multivariable (adjusted) logistic regression was used within the generalized estimating equations (GEE) framework to account for repeated measures within registrars. An exchangeable working correlation structure was assumed. Univariable analyses estimated the relationship of each covariate with the outcome. Covariates with a univariate P value <0.20 were considered for inclusion in the multivariable model.

Once the model with all significant covariates was fitted, model reduction was assessed. Covariates which were no longer significant (at p <0.2) in the multivariable model were tested for removal from the model. If the covariate’s removal did not substantively change the resulting model (defined as a change in the effect size (odds ratio) of less than 10%), the covariate was removed from the final model.

To address our aim of establishing associations of a problem/diagnosis being AD, three multivariable models were built, each with ‘AD’ as the outcome.

In the first model, patient, practice, and registrar independent variables, plus whether the problem/diagnosis was a new one, were included in the model to assess variables associated with a registrar encountering AD problems/diagnoses (compared to other problems/diagnoses).

In the second model, these patient/practice/registrar variables were modelled along with additional ‘consultation ’variables: consultation duration and number of problems/diagnoses dealt with in the consultation, and the registrar seeking information/ assistance (from their supervisor or from another source).

In the third model, all variables in the previous two models were included with further ‘consultation’ variables: pathology ordered, follow-up ordered, learning goals generated, referral ordered, and medication prescribed.

The rationale for conducting the successive regression models was that patient, registrar, and practice factors could plausibly influence whether a patient presents to the registrar with AD. Evaluation of these influences may be compromised by inclusion in the model of factors operating once the consultation is progressing. Similarly, evaluation of the content of the consultation may be compromised by the inclusion of actions arising from the consultation.

Analysis was performed at the level of problem/diagnosis. The regressions modelled the log-odds that a problem/diagnosis was classified as atopic dermatitis. Results are presented as odds ratios with 95% CI. Statistical analyses were conducted using STATA 14.1 (StataCorp, College Station, TX, USA) and SAS V9.4 (SAS Institute Inc., Cary, NC, USA). This project has been approved by The University of Newcastle Human Research Ethics Committee.

## Results

From 2010–2019, 2,783 registrars (96.1% response rate) provided data from 381,180 consultations, within which 595,412 problems were managed. The demographics of participating registrars are shown below (Table 1).

Of all problems managed 3,285 (0.6% [95% CI: 0.53 0.57]) were AD. Among AD problems/diagnoses, 34% were for a new diagnosis. Characteristics associated with a problem/ diagnosis being AD are presented here. (Table 2).

Results of univariate and multivariable logistic regression with the outcome of problem/diagnosis being AD are presented in (Table 3).

Statistically significant multivariable associations of an AD problem being seen were age 0–1 years (OR 1.80 [95% CI 1.60 2.02] compared to age 1 to 12 years) and patients being of non-English speaking background (OR 1.17 [95% CI: 1.02 1.34]). AD was less likely to be seen in Aboriginal or Torres Strait Islander patients (OR 0.66 [95% CI 0.47 0.93]). Patients presenting with AD were more likely to be new to the practice (OR 1.70 [95% CI 1.46 1.98]) or new to the registrar (OR 1.52 [95% CI 1.39 1.66]). AD was less likely to be seen by registrars working in outer regional, remote, or very remote areas (OR 0.79 [95%CI 0.66, 0.96] compared to major city locations).

AD was less likely to be a new problem for the patient (OR 0.20 [95% CI 0.18 0.22] compared with an existing problem) and was associated with more issues being dealt with in these consultations (OR 1.29 [ 95% CI 1.23 1.35]). Pathology was less likely to be ordered (OR 0.18 [95% CI 0.14 0.25]) and there was a strong association with medication being prescribed (OR 5.64 [95% CI 5.07 6.27]).

Learning goals were more likely to be generated for AD than other problems/diagnoses (OR 1.14 [95% CI 1.01 1.28]). AD problems/diagnoses were associated with seeking information or assistance, both from registrars’ supervisors (OR 1.59 [95% CI 1.38 1.84]) or from other sources (OR 1.54 [95% CI 1.37 1.73).

There were 3,185 prescriptions written for AD. Table 4 outlines the most prescribed medications and, where possible, the potency of the prescribed medication. Topical corticosteroids (TCS) were the most prescribed medicines. Of these, the most prescribed were hydrocortisone (mild/moderate potency), mometasone (potent), betamethasone (moderate/ potent), methylprednisolone aceponate (moderate), and triamcinolone (moderate). Antibiotics were prescribed in 6.5% of AD problems/diagnoses. Cephalexin, an oral antibiotic, was the most prescribed.

## Discussion

Factors associated with GP registrar exposure to management of AD have not been well-investigated. Specialist vocational training is an essential time for registrars to build exposure to, and confidence in managing, common primary care presentations. Given community prevalence and disease burden, AD exposure comprises an essential part of this experience.

### Registrar Experience of Patient Presentations with AD

Atopic dermatitis accounted for 0.6% of problems seen by Australian GP registrars. This is quite low when compared with the limited existing literature on GPs’ consultations with AD. In a study of UK GPs, 14% of consultations contained one or more dermatology problems/diagnoses (ICPC-2 defined) and 12% of these problems (the joint-highest of any particular skin condition) were AD [17].

AD is most common in early childhood and infancy [3]. Registrars in our study were more likely to see AD in patients aged 0–1 years compared to other age-groups, consistent with established peak periods of diagnosis [32]. Registrars in our study also see a younger patient demographic than established GPs [25], including in the peak 0–1 year age-group for AD [33]. Thus, the finding of low frequency of seeing patients with AD compared to established GPs is of particular interest.

Despite its greater prevalence in a younger population, AD is a chronic disease. Concerns have been previously raised regarding registrar exposure to chronic disease [19, 22, 24, 26]. Our findings suggest that the pattern of presentations for AD is consistent with other chronic diseases and that patients (and parents) may be more likely to choose to attend an established GP for management of this condition.

### Registrar Confidence Managing AD

Our findings suggest that the registrars’ modest levels of experience with AD in GP vocational training, in addition to deficits in undergraduate and hospital-based pre-vocational training [34–37], may limit confidence in its management. Registrars were more likely to generate learning goals within these consultations in addition to seeking information and assistance from both supervisors and other sources. Consistent with previous studies [21, 38], our findings again suggest that skin conditions remain challenging for registrars to manage.

AD being the problem seen is strongly associated with medication prescription. Our registrars prescribed a topical corticosteroid (TCS) in 82% of consultations. TCS comprise a first line treatment for AD [6, 39]. There are 4 classes of TCS according to potency [31], grouped from mild to very potent. Chidwick et al found that GPs were most likely to prescribe potent TCS [7]. In contrast, we found that registrars were most likely to prescribe TCS of mild or moderate potency. Mild to moderate potency TCS were prescribed in 54% of AD presentations. In particular, TCS containing hydrocortisone was most likely to be prescribed. This greater prescribing of lower potency TCS suggests that registrars may have some discomfort in prescribing potent steroids. This may be in part due to the young ages of the population seen with AD. However, this is also of significance given corticosteroid phobia has been named as a significant barrier for optimizing treatment in AD [31, 39, 40]. In addition, registrars prescribed antibiotics for AD in 6.5% of presentations. Bacterial colonization and superinfection may occur in AD, due to a compromised cutaneous barrier [6]. However, as shown by a recent randomized control trial, children with mildly infected AD do not require oral or topical antibiotic therapy [41]. Optimized management in these scenarios is through prophylactic measures such as the increased use of emollients and TCS [31, 41].

### Demographics of AD Presentations

We also found that location and specific patient demographics impacted upon registrar exposure to AD. Our findings have demonstrated a reduction in presentation of AD in outer regional and remote areas. This contrasts with previous findings of no significant differences between urban and rural areas in the prevalence of AD [42]. Barriers to access healthcare in regional and remote areas may account for this finding. Availability of appointments, and geographic distances involved, may lead to other health concerns being prioritized above AD for management. The context of this finding is a tendency for the impact of skin problems compared to other health conditions to be underestimated by GPs (and, sometimes, for this perspective to be understood by patients themselves)[43].

We identified an increase in AD presenting in patients from NESB backgrounds. AD rates have been shown to vary between different ethnic groups [44, 45], and is notably more predominant in high income countries [45]. In Australia, AD has been shown to be increased in children of Chinese migrants [44, 46] however there are varied reported rates for AD in children of other ethnic migrant descent [46, 47]. Whether children are first- or second-generation migrants has also been suggested to impact upon atopic disease prevalence [48]. Other factors including the timing and age of migration and duration of residence may also impact AD risk [48].

Our results also showed a reduction of AD presentation in Aboriginal and Torres Strait Islander patients. This finding was statistically significant and noteworthy particularly in the context of limited evidence around the prevalence of AD in Aboriginal and Torres Strait Islander patients [49].

Interpretation of our findings of demographic associations must be cautious, however. Further research is required to establish the relative influences of AD prevalence, relative access to health care, and other factors in associations of rurality, socioeconomic status, identification as Aboriginal or Torres Strait Islander, and Non-English-Speaking Background with AD presentations to registrars.

### Strengths and Limitations

Strengths of this study include a large data set (595,412 data points) and its high response rate (96.1% - particularly high for a study of GPs[50]). Findings are generalizable across Australia, and potentially internationally, given the broad coverage of Australian regions distributed across urban, rural, remote, and very remote classifications.

A limitation of this study is that we were unable to comprehensively assess TCS prescriptions concentration as this information is not available in the data. Another limitation was not being able to assess severity of AD seen by registrars. This would have provided valuable information in interpreting steroid and management choice.

There is some difficulty in interpreting TCS choice further given that potency depends on concentration and formulation. For example, TCS containing betamethasone was prescribed in 22% of TCS prescriptions. This medication may be formulated to be moderate, potent, or very potent, and within this study we were unable to establish potency of how this medication was prescribed.

### Implications for Registrar Education

Our findings show that GP registrars may be exposed to managing AD less frequently than their more senior GP counterparts. As such, there may be some areas in which registrars may lack confidence in management. An example is TCS choice and optimal use of antibiotics. Limited exposure could also limit experience in developing the nuanced clinical skills in shared decision-making and patient self-management required in AD.[17, 51] These areas could be addressed in registrars’ education programs. Our findings also show that geographic and population factors may impact registrar exposure to clinical experience with AD. RTO-wide education programs should take this variability into account.

## Conclusion

Our findings show that GP registrars encounter AD less often than their more senior counterparts and this experience may be variable depending on rurality and region of practice. We have identified evidence that registrars may find AD challenging to manage and that there may be some gaps in management knowledge and application. Registrar education programs should address these gaps.

## Figures and Tables

**Table 1:** Registrar and Practice Variables for Atopic Dermatitis Problems Being Seen

Registrar variables (n=2783)		n (%)
Registrar gender	Female	1728 (62.1)
Qualified as doctor (primary medical degree) in Australia	Yes	547 (19.8)
Pathway registrar enrolled in	General	1930 (70.0)
**Registrar round/practice variables (n=6414)**		
Registrar age (years)	Mean ± SD	32.6 (6.3)
Registrar works PT	Yes	1420 (22.9)
Registrar training term	Term 1	2640 (41.2)
	Term 2	2091 (32.6)
	Term 3	1683 (26.2)
Practice rurality	Major city	3983 (62.7)
	Inner regional	1633 (25.7)
	Outer regional	653 (10.3)
	Remote	64 (1.0)
	Very remote	16 (0.3)
Practice SEIFA index	Mean ± SD	5.5 (2.8)
Practice routinely bulk bills	Yes	1784 (28.1)
Registrar worked at practice previously	Yes	1343 (21.2)
Practice Size	Small (1–5 GPs)	2371 (38.4)
	Large (6–10+ GPs)	3811 (61.6)

**Table 2:** Characteristics Associated with Seeing a Patient with Atopic Dermatitis

			Atopic dermatitis		
Factor group	Variable	Class	No	Yes	p
Patient factors	Patient age group	0–1 years	24215 (4%)	687 (21%)	<0.001
		2–12years	48591 (8%)	901 (28%)	
		13–24 years	77871 (13%)	591 (18%)	
		25–44 years	160401 (28%)	579 (18%)	
		45+ years	271596 (47%)	471 (15%)	
	Patient gender	Male	221923 (38%)	1411 (44%)	<0.001
		Female	356223 (62%)	1798 (56%)	
	Aboriginal and Torres Strait Islander	No	540148 (98%)	3036 (99%)	0.038
		Yes	10055 (2%)	39 (1%)	
	NESB*	No	505865 (91%)	2776 (90%)	0.014
		Yes	48101 (9%)	315 (10%)	
	Patient/practice status	Existing patient	242797 (42%)	1097 (34%)	<0.001
		New to registrar	291926 (51%)	1833 (57%)	
		New to practice	43213 (7%)	292 (9%)	
Registrar factors	Registrar gender	Male	216382 (37%)	1200 (37%)	0.999
		Female	375745 (63%)	2085 (63%)	
	Registrar FT or PT**	Part-time	133911 (23%)	790 (25%)	0.17
		Full-time	438268 (77%)	2405 (75%)	
	Term	Term 1	248656 (42%)	1288 (39%)	<0.001
		Term 2	190762 (32%)	1193 (36%)	
		Term 3	152709 (26%)	804 (24%)	
	Worked at practice previously	No	458411 (78%)	2563 (79%)	0.63
		Yes	126188 (22%)	671 (21%)	
	Qualified as doctor in Australia	No	108879 (18%)	532 (16%)	0.006
		Yes	480721 (82%)	2739 (84%)	
	Registrar age	mean (SD)	33 (6)	32 (6)	0.010
Practice factors	Practice size	Small	221940 (39%)	1145 (36%)	0.007
		Large	349327 (61%)	2044 (64%)	
	Practice routinely bulk bills	No	423524 (72%)	2283 (71%)	0.10
		Yes	163125 (28%)	948 (29%)	
	Rurality	Major city	364641 (62%)	2239 (69%)	<0.001
		Inner regional	150534 (26%)	726 (22%)	
		Outer regional remote	70686 (12%)	274 (8%)	
	Region	Region 1	135287 (23%)	546 (17%)	<0.001
		Region 2	36043 (6%)	153 (5%)	
		Region 3	62968 (11%)	396 (12%)	
		Region 4	198987 (34%)	1397 (43%)	
		Region 5	10381 (2%)	31 (0.9%)	
		Region 6	97271 (16%)	550 (17%)	
		Region 7	51190 (9%)	212 (6%)	
	SEIFA index***	mean (SD)	5 (3)	6 (3)	<0.001
Consultation factors	New problem seen	No	238042 (44%)	1957 (66%)	<0.001
		Yes	302743 (56%)	989 (34%)	
	Sought help any source	None	487113 (82%)	2505 (76%)	<0.001
		Supervisor	43925 (7%)	319 (10%)	
		Other sources	61089 (10%)	461 (14%)	
	Pathology ordered	No	490393 (83%)	3215 (98%)	<0.001
		Yes	101734 (17%)	70 (2%)	
	Follow-up ordered	None	333581 (56%)	2094 (64%)	<0.001
		GP appt or phone	225508 (38%)	1128 (34%)	
		With someone else	33037 (6%)	63 (2%)	
	Learning goals generated	No	452325 (82%)	2467 (80%)	0.032
		Yes	100596 (18%)	604 (20%)	
	Referral ordered	No	518160 (88%)	3063 (93%)	<0.001
		Yes	73967 (12%)	222 (7%)	
	Medication prescribed	No	337749 (57%)	646 (20%)	<0.001
		Yes	254378 (43%)	2639 (80%)	
	Consultation duration	mean (SD)	19 (10)	17 (8)	<0.001
	Number of problems	mean (SD)	2 (1)	2 (1)	<0.001

NESB = Non-English-Speaking Background; (PT)=Part Time, (FT)= Full Time, SEIFA = Socio-Economic Indexes for Areas

**Table 3:** Simple and Adjusted Logistic Regression With Outcome: Problem/Diagnosis is Atopic

			Univariable		Multivariable	
Factor group	Variable	Class	OR [95% CI]	p	OR [95% CI]	P
*Patient factors*	Patient age group	0–1 years	1.53 (1.38, 1.70)	<.001	1.80 (1.60, 2.02)	<.001
	Referent: 2–12 years	13–24 years	0.41 (0.37, 0.45)	<.001	0.34 (0.30, 0.38)	<.001
		25–44 years	0.19 (0.17, 0.22)	<.001	0.15 (0.13, 0.17)	<.001
		45+ years	0.09 (0.08, 0.11)	<.001	0.06 (0.05, 0.07)	<.001
	Aboriginal and Torres Strait Islander	Yes	0.72 (0.53, 0.98)	0.038	0.66 (0.47, 0.93)	0.016
	NESB	Yes	1.17 (1.03, 1.33)	0.014	1.17 (1.02, 1.34)	0.022
	Patient/practice status	New to registrar	1.38 (1.27, 1.49)	<.001	1.52 (1.39, 1.66)	<.001
	Referent: Existing patient	New to practice	1.49 (1.31, 1.69)	<.001	1.70 (1.46, 1.98)	<.001
*Registrar factors*	Term	Term 2	1.21 (1.11, 1.32)	<.001	1.20 (1.09, 1.32)	<.001
	Referent: Term 1	Term 3	1.01 (0.92, 1.11)	0.81	1.05 (0.94, 1.16)	0.40
*Practice factors*	Rurality	Inner regional	0.78 (0.71, 0.86)	<.001	0.88 (0.77, 1.00)	0.053
	Referent: major city	Outer regional or remote	0.63 (0.55, 0.72)	<.001	0.79 (0.66, 0.96)	0.015
	Region Referent: Region 1	Region 2	1.05 (0.86, 1.29)	0.62	1.03 (0.83, 1.28)	0.78
		Region 3	1.55 (1.33, 1.81)	<.001	1.78 (1.48, 2.13)	<.001
		Region 4	1.74 (1.55, 1.95)	<.001	1.46 (1.28, 1.66)	<.001
		Region 5	0.74 (0.52, 1.07)	0.11	0.83 (0.54, 1.27)	0.39
		Region 6	1.39 (1.22, 1.60)	<.001	1.13 (0.96, 1.33)	0.14
		Region 7	1.02 (0.86, 1.21)	0.83	1.06 (0.87, 1.29)	0.54
*Consultation factors*	New problem seen	Yes	0.39 (0.36, 0.42)	<.001	0.20 (0.18, 0.22)	<.001
	Sought help any source	Other sources	1.50 (1.35, 1.66)	<.001	1.54 (1.37, 1.73)	<.001
	Referent: None	Supervisor	1.41 (1.25, 1.60)	<.001	1.59 (1.38, 1.84)	<.001
	Consultation duration		0.98 (0.97, 0.98)	<.001	0.98 (0.97, 0.98)	<.001
	Number of problems		0.89 (0.86, 0.92)	<.001	1.29 (1.23, 1.35)	<.001
	Pathology ordered	Yes	0.11 (0.08, 0.13)	<.001	0.18 (0.14, 0.25)	<.001
	Follow-up ordered	GP appt or phone	0.79 (0.73, 0.86)	<.001	0.94 (0.85, 1.03)	0.19
	Referent: None	With someone else	0.30 (0.24, 0.39)	<.001	0.30 (0.22, 0.40)	<.001
	Learning goals generated	Yes	1.11 (1.01, 1.22)	0.032	1.14 (1.01, 1.28)	0.037
	Referral ordered	Yes	0.51 (0.45, 0.58)	<.001	0.65 (0.55, 0.77)	<.001
	Medication prescribed	Yes	5.48 (5.00, 6.01)	<.001	5.64 (5.07, 6.27)	<.001

**Table 4:** Medications Prescribed for Atopic Dermatitis Presentations

Medication type	Number of Prescriptions (n = 3185)

**Topical Corticosteroids**	**2676**
Hydrocortisone aceponate (mild)	781
Mometasone (potent)	614
Methylprednisolone aceponate (moderate)	572
Betamethasone (moderate/potent)	571
Triamcinolone (moderate)	125
Clobetasol (very potent)	4
Unspecified	9
**Antibiotics**	**215**
**Topical antifungal**	**77**
**Topical Calcineurin Inhibitor**	**67**
**Immunosuppressants and immunomodulators**	**49**
Prednisolone	46
Azothioprine	3
**Antihistamine**	**39**
**Emollients and antimicrobial measures**	**31**
**Other**	**31**

## References

[1] Weidinger S, Novak N (2016). Atopic dermatitis. Lancet.

[2] Correa MC, Nebus J (2012). Management of patients with atopic dermatitis: the role of emollient therapy. Dermatol Res Pract.

[3] Kay J, Gawkrodger DJ, Mortimer MJ, Jaron AG (1994). The prevalence of childhood atopic eczema in a general population. J Am Acad Dermatol.

[4] Asher MI, Montefort S, Bjorksten B, Lai CK, Strachan DP, Weiland SK (2006). Worldwide time trends in the prevalence of symptoms of asthma, allergic rhinoconjunctivitis, and eczema in childhood: ISAAC Phases One and Three repeat multicountry cross-sectional surveys. Lancet.

[5] Mortz CG, Andersen KE, Dellgren C, Barington T, Bindslev-Jensen C (2015). Atopic dermatitis from adolescence to adulthood in the TOACS cohort: prevalence, persistence and comorbidities. Allergy.

[6] Harris VR, Cooper AJ (2017). Atopic dermatitis: the new frontier. Med J Aust.

[7] Chidwick K, Busingye D, Pollack A, Osman R, Yoo J, Blogg S (2020). Prevalence, incidence and management of atopic dermatitis in Australian general practice using routinely collected data from MedicineInsight. Australas J Dermatol.

[8] Hay RJ, Johns NE, Williams HC, Bolliger IW, Dellavalle RP, Margolis DJ (2014). The global burden of skin disease in 2010: an analysis of the prevalence and impact of skin conditions. J Invest Dermatol.

[9] Drucker AM, Wang AR, Li WQ, Sevetson E, Block JK, Qureshi AA (2017). The Burden of Atopic Dermatitis: Summary of a Report for the National Eczema Association. J Invest Dermatol.

[10] Beattie PE, Lewis-Jones MS (2006). A comparative study of impairment of quality of life in children with skin disease and children with other chronic childhood diseases. Br J Dermatol.

[11] Faught J, Bierl C, Barton B, Kemp A (2007). Stress in mothers of young children with eczema. Arch Dis Child.

[12] Su JC, Kemp AS, Varigos GA, Nolan TM (1997). Atopic eczema: its impact on the family and financial cost. Arch Dis Child.

[13] Eichenfield LF, Boguniewicz M, Simpson EL, Russell JJ, Block JK, Feldman SR (2015). Translating Atopic Dermatitis Management Guidelines Into Practice for Primary Care Providers. Pediatrics.

[14] Ballardini N, Kull I, Soderhall C, Lilja G, Wickman M, Wahlgren CF (2013). Eczema severity in preadolescent children and its relation to sex, filaggrin mutations, asthma, rhinitis, aggravating factors and topical treatment: a report from the BAMSE birth cohort. Br J Dermatol.

[15] Foley P, Zuo Y, Plunkett A, Marks R (2001). The frequency of common skin conditions in preschool-age children in Australia: atopic dermatitis. Arch Dermatol.

[16] Atopic Dermatitis. eTG Complete.

[17] Le Roux E, Edwards PJ, Sanderson E, Barnes RK, Ridd MJ (2020). The content and conduct of GP consultations for dermatology problems: a cross-sectional study. Br J Gen Pract.

[18] Mulquiney KJ, Tapley A, van Driel ML, Morgan S, Davey AR, Henderson KM (2018). Referrals to dietitians/nutritionists: A cross-sectional analysis of Australian GP registrars’ clinical practice. Nutr Diet.

[19] Gordon J, Harrison C, Miller G (2016). General practitioners and general practice registrars: A comparison of clinical activity. Aust Fam Physician.

[20] Thomson JS, Anderson KJ, Mara PR, Stevenson AD (2011). Supervision--growing and building a sustainable general practice supervisor system. Med J Aust.

[21] Whiting G, Magin P, Morgan S, Tapley A, Henderson K, Oldmeadow C (2017). General practice trainees’ clinical experience of dermatology indicates a need for improved education: A cross-sectional analysis from the Registrar Clinical Encounters in Training Study. Australas J Dermatol.

[22] De Jong J, Visser MR, Wieringa-de Waard M (2011). Exploring differences in patient mix in a cohort of GP trainees and their trainers. BMJ Open.

[23] Morgan S, Henderson KM, Tapley A, Scott J, Spike NA, Laurence CO, van Driel ML, Thomson AM, Magin PJ Problems Managed by Australian General Practice Registrars: Results from the Re-CEnT (Registrar Clinical Encounters in Training) Study. 2014. Education for Primary Care.

[24] de Jong J, Visser MR, Mohrs J, Wieringa-de Waard M (2011). Opening the black box: the patient mix of GP trainees. Br J Gen Pract.

[25] Bonney A, Morgan S, Tapley A, Henderson K, Holliday E, Davey A, van Driel M, Spike N, Regan C, Magin P Older patients’ consultations with general practice trainees: a cross-sectional study. 2017. Australasian Journal on Ageing.

[26] Magin P, Morgan S, Henderson K, Tapley A, McElduff P, Pearlman J (2014). Family medicine trainees’ clinical experience of chronic disease during training: a cross-sectional analysis from the registrars’ clinical encounters in training study. BMC Med Educ.

[27] Britt H (1997). A new coding tool for computerised clinical systems in primary care--ICPC plus. Aust Fam Physician.

[28] Taylor R, Clarke L, Edwards D (2018). National report on the 2018 National Registrar Survey.

[29] Australian Statistical Geographical Classification - Remoteness Area.

[30] (2016). Census of Population and Housing: Socio-Economic Indexes for Areas (SEIFA), Australia.

[31] Mooney E, Rademaker M, Dailey R, Daniel BS, Drummond C, Fischer G (2015). Adverse effects of topical corticosteroids in paediatric eczema: Australasian consensus statement. Australas J Dermatol.

[32] Langan SM, Irvine AD, Weidinger S (2020). Atopic dermatitis. Lancet.

[33] Britt H, Miller G, Bayram C (2016). A decade of Australian general practice activity 2006–07 to 2015–16.

[34] Wearne SM, Magin PJ, Spike NA (2018). Preparation for general practice vocational training: time for a rethink. Med J Aust.

[35] Gupta A, Chong AH, Scarff CE, Huilgol SC (2017). Dermatology teaching in Australian Medical Schools. Australas J Dermatol.

[36] Singh DG, Boudville N, Corderoy R, Ralston S, Tait CP (2011). Impact on the dermatology educational experience of medical students with the introduction of online teaching support modules to help address the reduction in clinical teaching. Australas J Dermatol.

[37] Sladden MJ, Graham-Brown RA (1989). How many GP referrals to dermatology outpatients are really necessary?. J R Soc Med.

[38] Willems A, Fielding A, Tng V, Holliday E, van Driel M, Ball J, Davey A, FitzGerald K, Spike N, Magin P (2021). General Practice Registrars’ management of and specialist referral patterns for atopic dermatitis: General Practice registrar management of atopic dermatitis. Dermatol Pract Concept.

[39] Eichenfield LF, Tom WL, Berger TG, Krol A, Paller AS, Schwarzenberger K (2014). Guidelines of care for the management of atopic dermatitis: section 2. Management and treatment of atopic dermatitis with topical therapies. J Am Acad Dermatol.

[40] Smith SD, Harris V, Lee A, Blaszczynski A, Fischer G (2017). General practitioners knowledge about use of topical corticosteroids in paediatric atopic dermatitis in Australia. Aust Fam Physician.

[41] Francis NA, Ridd MJ, Thomas-Jones E, Butler CC, Hood K, Shepherd V (2017). Oral and Topical Antibiotics for Clinically Infected Eczema in Children: A Pragmatic Randomized Controlled Trial in Ambulatory Care. Ann Fam Med.

[42] Ferrandiz-Mont D, Wahyuniati N, Chen HJ, Mulyadi M, Zanaria TM, Ji DD (2018). Hygiene practices: Are they protective factors for eczema symptoms?. Immun Inflamm Dis.

[43] Magin PJ, Adams J, Heading GS, Pond CD (2009). Patients with skin disease and their relationships with their doctors: a qualitative study of patients with acne, psoriasis and eczema. Med J Aust.

[44] Martin PE, Koplin JJ, Eckert JK, Lowe AJ, Ponsonby AL, Osborne NJ (2013). The prevalence and socio-demographic risk factors of clinical eczema in infancy: a population-based observational study. Clin Exp Allergy.

[45] Odhiambo JA, Williams HC, Clayton TO, Robertson CF, Asher MI, Group IPTS (2009). Global variations in prevalence of eczema symptoms in children from ISAAC Phase Three. J Allergy Clin Immunol.

[46] Mar A, Tam M, Jolley D, Marks R (1999). The cumulative incidence of atopic dermatitis in the first 12 months among Chinese, Vietnamese, and Caucasian infants born in Melbourne, Australia. J Am Acad Dermatol.

[47] Ponsonby AL, Glasgow N, Pezic A, Dwyer T, Ciszek K, Kljakovic M (2008). A temporal decline in asthma but not eczema prevalence from 2000 to 2005 at school entry in the Australian Capital Territory with further consideration of country of birth. Int J Epidemiol.

[48] Tham EH, Loo EXL, Zhu Y, Shek LP (2019). Effects of Migration on Allergic Diseases. Int Arch Allergy Immunol.

[49] Heyes C, Tait C, Toholka R, Gebauer K (2014). Non-infectious skin disease in Indigenous Australians. Australas J Dermatol.

[50] Bonevski B, Magin P, Horton G, Foster M, Girgis A (2011). Response rates in GP surveys - trialling two recruitment strategies. Aust Fam Physician.

[51] Ridd MJ, King AJL, Le Roux E, Waldecker A, Huntley AL (2017). Systematic review of self-management interventions for people with eczema. Br J Dermatol.

